# A Case of Malaria Predisposing to *Salmonella* Bacteremia in a Returning Traveler from Nigeria

**DOI:** 10.1155/2018/8463417

**Published:** 2018-09-27

**Authors:** Robert Jakubowski, Lisa L. Steed, Susan E. Dorman, Camelia Marculescu

**Affiliations:** ^1^Division of Infectious Diseases, Department of Medicine, Medical University of South Carolina, 135 Rutledge Avenue, MSC 752, Charleston, SC, USA; ^2^Department of Pathology and Laboratory Medicine, Medical University of South Carolina, 171 Ashley Avenue, MSC 908, Charleston, SC, USA

## Abstract

We describe a febrile adult returning to the U.S. from Nigeria. Malaria was diagnosed by rapid antigen testing, but recognition of invasive nontyphoidal *Salmonella* disease was delayed. While the moniker, “typhomalaria,” once used to describe an illness with features of malaria and typhoid fever, has fallen out of favor, it may nevertheless be a helpful reminder to clinicians that both infectious diseases can arise in the same patient. Blood cultures should be obtained routinely in febrile returning travelers from malaria-endemic regions, including those in whom the diagnosis of malaria has already been established.

## 1. Introduction

The moniker “typhomalaria” first appeared in the medical literature in 1876 when U.S. Army surgeon J. J. Woodward used it to describe a febrile illness that had been observed among U.S. Civil War soldiers and had features of typhoid as well as periodic chills and rigors [1, 2]. At that time, the parasitic etiology of malaria was only on the verge of discovery [3]. Nevertheless, Woodward correctly concluded based on postmortem examinations of intestinal ulcers and serum pigment granules that typhomalaria was caused by “two varieties of miasmata,” typhoid fever and a malarial disease [4]. In 1929, Dr. George Giglioli, working in British Guiana, noticed an association between *Salmonella* paratyphi C and malaria. He observed that paratyphoid C was more prevalent and severe during malaria outbreaks [5]. Malaria was largely eradicated from the U.S. by 1949, and typhomalaria has since faded from the medical lexicon [6]. In this case report, we present a case of concomitant malaria and invasive nontyphoidal *Salmonella* infection in a returning traveler to the U.S.

## 2. Case Presentation

A 50-year-old female returning traveler presented to an emergency room in South Carolina for evaluation of syncope. Thirteen days prior she had returned from a 10-day trip to Lagos, Nigeria, where she visited family. While in Lagos, she felt well. Six days after returning to the U.S., she developed nausea and diarrhea. Three days later, she developed fevers and sweats that occurred multiple times daily. Twelve days after returning to the U.S., she experienced two episodes of syncope and then sought medical care. She had no headache, neck stiffness, sore throat, or respiratory symptoms. She reported that while in Lagos, she stayed in a home in an urban environment. She used mosquito repellent with N, N-diethyl-meta-toluamide (DEET), and slept indoors with closed windows but no mosquito net. She did not recall any mosquito bites. While in Lagos, she ate food prepared in the home after purchase from a local grocery store, and she drank only bottled water. She did not recall having been in contact with anyone who was sick while she was in Lagos. She reported having taken a prescription medication for malaria prevention, but we were unable to verify this. She had received oral typhoid, yellow fever, hepatitis A, TDaP, influenza, and meningococcal vaccines within the preceding year. The recent trip was her third to Nigeria; she had been well during and after her previous two trips. She was born and raised in South Carolina and had no other international travel. She had no significant past medical history and was not taking any medications at the time of presentation. She worked as a manager at a retail clothing store.

On examination at presentation, the temperature was 100.8°, pulse 117/minute and regular, blood pressure 127/78, respirations 18/minute and unlabored, and O_2_ saturation was 99% breathing ambient air. She appeared comfortable, well-nourished, and not chronically ill. There was no jaundice or lymphadenopathy. Lungs were clear. Heart sounds were normal aside from tachycardia, and there was no murmur. The abdomen was not tender, and there was no hepatosplenomegaly. She was alert, and neurologic exam was unremarkable. Laboratory test results are shown in Table 1.

There was no hemoglobin detected in the urine. Blood cultures were drawn. Thick and thin blood smears were prepared and Giemsa-stained. Microscopic examination showed a microcytic normochromic anemia, numerous erythrocytes with trophozoite rings, and rare banana gametocytes characteristic of *P. falciparum* malaria. Parasitemia was 3.5%. Rapid malaria diagnostic testing using the BinaxNOW® Malaria test (Alere Inc., Waltham Massachusetts) was positive for *P. falciparum*. An ARCHITECT HIV Ag/Ab Combo (Abbott, Wiesbaden, Germany) test was negative. Nucleic acid amplification-based testing of a stool specimen was negative for adenovirus, *Campylobacter*, *Cyclospora cayetanensis*, *Clostridium difficile*, enteroaggregative *Escherichia coli*, enteropathogenic *E. coli*, enterotoxigenic *E. coli*, Shiga-like toxin producing *E. coli*, *Shigella*, enteroinvasive *E. coli*, *Entamoeba histolytica*, *Giardia lamblia*, human astrovirus, norovirus GI/GII, *Plesiomonas shigelloides*, rotavirus A, *Salmonella*, sapovirus, *Vibrio*, *Vibrio cholera*, and *Yersinia enterocolitica* (FilmArray™ Gastrointestinal Panel, BioFire Diagnostics LLC, Salt Lake City, UT). At 48 hours of incubation, blood cultures obtained at presentation grew gram-negative rods identified as a *Salmonella* species by MALDI-TOF mass spectrometry. A triple sugar iron agar slant showed a nontyphoidal *Salmonella* phenotype. The isolate was identified as *Salmonella enterica* subspecies *enterica* serovar *stanleyville* (1,4,12,27:z4,z23:[1,2]) by the South Carolina Department of Health and Environmental Control Public Health Laboratory using serotyping with specific O and H antisera according to the Kaufman–White scheme. She was treated for uncomplicated *P. falciparum* malaria with oral doxycycline and quinidine for 7 days. Fevers resolved by day 3 at the hospital and repeat blood smear remained negative for malaria 16 days later. For nontyphoidal *Salmonella* bacteremia, she was treated with ceftriaxone for 2 weeks; diarrhea and all other signs/symptoms resolved within 10 days.

## 3. Discussion

We report the occurrence of concomitant *P. falciparum* malaria and nontyphoidal *Salmonella* bacteremia in a traveler returning from Nigeria. Each of these infectious diseases is a major cause of morbidity and mortality in sub-Saharan Africa. Nigeria bears the majority of the worlds' burden of malaria, accounting for 27% of cases globally—in 2016, there were an estimated 57 million malaria cases and 100,700 deaths, and over 85% of cases were due to *P. falciparum* [7, 8]. Invasive salmonellosis mostly affects young children, those with severe malnutrition, HIV, or sickle cell anemia [9, 10]. A systematic review showed that among adults in Africa, 42% of (nonmalaria) bloodstream infections were due to *Salmonella* enterica, mostly nontyphoidal *Salmonella* [11]. In Nigeria, one-quarter to one-half of all bacteremias have been shown to be due to *Salmonella enterica*, about half of which are due to nontyphoidal *Salmonella* [12, 13].

Co-infection with malaria and *Salmonella* has been recognized for some time and is expected based on the overlapping geographical distribution of these infectious diseases [14, 15]. The epidemiology of malaria-*Salmonella *co-infections is limited by a lack of good surveillance data on invasive salmonellosis in sub-Saharan Africa. Overall, there is inconsistent access to culture and molecular techniques outside of vaccination trials [16]. However, a meta-analysis of over 11,000 patients (adults and children) in Africa showed that 6.5% of patients with malaria had fungal or bacterial bloodstream co-infections [11]. Co-infection was initially attributed to the parallel rise of both organisms during the rainy season. However, a study in Tanzania controlled for precipitation by comparing two locations with similar rainy seasons and contrasting malaria prevalence [17]. In children admitted with fever, bacteremia of any etiology was about twice as common (9.2% vs. 4.3%) in the area with high malarial burden, but nontyphoidal *Salmonella* isolates were almost ten times more common (48.2% vs. 5%) in the area with high malarial burden [17]. The same parallel rise in co-infection was seen in Gambian children during the rainy season. However, stool carriage of nontyphoidal *Salmonella* did not share the seasonal pattern leading authors to conclude that malaria predisposed to invasive nontyphoidal *Salmonella* infection [14].

The immunosuppressive effects of malaria have been well documented since the early 1970s. Recent work has shed light on a pathophysiologic mechanism for malaria as a risk factor for *Salmonella* bacteremia. This is through the adaptive phenomenon of immune tolerance, which limits self harm in response to the parasite. Malaria infection impairs the production proinflammatory cytokines by dendritic cells by inducing a state of refractory TLR signaling in favor of a downregulatory T cell response [18, 19]. In addition, in 2012, Cunnington et al. showed that malaria-induced hemolysis specifically suppressed the oxidative burst capacity of neutrophils, thereby allowing for sustained *Salmonella* bacterial replication in circulating, but functionally impaired, neutrophils in addition to mononuclear phagocytes [20]. Together, these hamper what normally would be a robust response by phagocytic cells to the bacteria facilitating their dissemination.

Awareness of the association between malaria and *Salmonella* bacteremia is important for clinicians. In patients with malaria, undiagnosed *Salmonella* bacteremia may lead to morbidity due to untreated salmonellosis and/or changes in antimalarial treatment if fevers are incorrectly attributed to malaria drug resistance. Thus, the moniker “typhomalaria” might be a useful reminder for clinicians that both infectious diseases can coexist in the same patient. Blood cultures should be obtained routinely in all febrile returning travelers from malaria-endemic regions, including those in whom the diagnosis of malaria has already been established.

## Figures and Tables

**Table 1:** 

Variable	Hospital day 1
White cell count	8500/*μ*L
Hemoglobin	10.7 gm/dL
Platelet count	142,000/*μ*L
Sodium	123 mmol/L
Potassium	3.9 mmol/L
Chloride	90 mmol/L
Bicarbonate	24 mmol/L
Blood urea nitrogen	16 mg/dL
Creatinine	1.0 mg/dL
Glucose	107 mg/dL
Calcium	7.7 mg/dL
Total protein	6.7 g/dL
Albumin	2.3 g/dL
AST	49 U/L
ALT	52 U/L
Alkaline phosphatase	111 U/L
Total bilirubin	2.4 mg/dL

## References

[1] Billings J. (1886). *Memoir of Joseph Janvier Woodward, 1833–1884*.

[2] Steele C. (1880). Was it typhomalarial fever?. *Western Lancet*.

[3] CDC (2018). *Malaria-About Malaria-History-Laveran and the Discovery of the Malaria Parasite*.

[4] Smith D. (1982). The rise and fall of typhomalarial fever: i. origins. *Journal of the History of Medicine and Allied Sciences*.

[5] Giglioli G. (1929). Paratyphoid C, an endemic disease in British Guiana. *Journal of Hygiene*.

[6] CDC (2018). *Malaria-About Malaria-History-Elimination of Malaria in the United States (1947-1951)*.

[7] World Health Organization (2017). *World Malaria Report 2017*.

[8] CDC (2018). *Malaria-Travelers-Malaria Information and Prophylaxis, by Country*.

[9] Brent A., Oundo J., Mwangi I., Ochola L., Lowe B., Berkley J. (2006). Salmonella Bacteremia in Kenyan Children. *Pediatric Infectious Disease Journal*.

[10] Morpeth S., Ramadhani H., Crump J. (2009). Invasive non‐typhisalmonelladisease in Africa. *Clinical Infectious Diseases*.

[11] Reddy E., Shaw A., Crump J. (2010). Community-acquired bloodstream infections in Africa: a systematic review and meta-analysis. *The Lancet Infectious Diseases*.

[12] Akinyemi K., Bamiro B., Coker A. (2007). Salmonellosis in Lagos, Nigeria: incidence of plasmodium falciparum-associated co-infection, patters of antimicrobial resistance, and emergence of reduced susceptibility to fluoroquinolones. *Journal of Health, Population and Nutrition*.

[13] Obaro S., Hassan-Hanga F., Olateju E. (2015). Salmonella bacteremia among children in central and northwest Nigeria, 2008-2015. *Clinical Infectious Diseases*.

[14] Mabey D., Brown A., Greenwood B. (1987). Plasmodium falciparum malaria and salmonella infections in Gambian children. *Journal of Infectious Diseases*.

[15] Bronzan R., Taylor T., Mwenechanya J. (2007). Bacteremia in Malawian children with severe malaria: prevalence, etiology, HIV coinfection, and outcome. *Journal of Infectious Diseases*.

[16] Feasey N., Dougan G., Kingsley R., Heyderman R., Gordon M. (2012). Invasive non-typhoidal salmonella disease: an emerging and neglected tropical disease in Africa. *The Lancet*.

[17] Biggs H., Lester R., Nadjm B. (2013). Invasive salmonella infections in areas of high and low malaria transmission intensity in Tanzania. *Clinical Infectious Diseases*.

[18] Perry J. A., Olver C., Burnett R., Avery A. (2005). Cutting edge: the acquisition of TLR tolerance during malaria infection impacts T cell activation. *Journal of Immunology*.

[19] Roux C., Butler B., Chau J. (2010). Both hemolytic anemia and malaria parasite-specific factors increase susceptibility to nontyphoidal *Salmonella enterica* serovar typhimurium infection in mice. *Infection and Immunity*.

[20] Cunnington A., de Souza J., Walther M., Riley E. (2011). Malaria impairs resistance to Salmonella through heme- and heme oxygenase–dependent dysfunctional granulocyte mobilization. *Nature Medicine*.

